# Surface Properties of Polymer Resins Fabricated with Subtractive and Additive Manufacturing Techniques

**DOI:** 10.3390/polym13234077

**Published:** 2021-11-24

**Authors:** Amal S. Al-Qahtani, Huda I. Tulbah, Mashael Binhasan, Maria S. Abbasi, Naseer Ahmed, Sara Shabib, Imran Farooq, Nada Aldahian, Sidra S. Nisar, Syeda A. Tanveer, Fahim Vohra, Tariq Abduljabbar

**Affiliations:** 1 Department of Prosthetic Dental Sciences, College of Dentistry, King Saud University, P.O. Box 21069, Riyadh 11475, Saudi Arabia; aalkahtany@ksu.edu.sa (A.S.A.-Q.); htulba@ksu.edu.sa (H.I.T.); fvohra@ksu.edu.sa (F.V.); 2 Department of Restorative Dentistry, Operative Division, College of Dentistry, King Saud University, P.O. Box 21069, Riyadh 11475, Saudi Arabia; mbinhasan@ksu.edu.sa (M.B.); sashabib@ksu.edu.sa (S.S.); n.aldahian@gmail.com (N.A.); 3 Department of Prosthodontics, Altamash Institute of Dental Medicine, Karachi 75500, Pakistan; maria_shakoor@hotmail.com (M.S.A.); naprosthodontist@gmail.com (N.A.); 4 Faculty of Dentistry, University of Toronto, Toronto, ON M5G 1G6, Canada; imran.farooq@mail.utoronto.ca; 5 Department of Operative Dentistry, Dow International Dental College, Dow University of Health Sciences, Karachi 74200, Pakistan; sidra.sadaf@duhs.edu.pk (S.S.N.); abeerah.tanveer@duhs.edu.pk (S.A.T.)

**Keywords:** CAD-CAM, 3D printing, interim polymers, dental material, roughness, flexural strength

## Abstract

This study aimed to compare the surface roughness, hardness, and flexure strength of interim indirect resin restorations fabricated with CAD-CAM (CC), 3D printing (3D), and conventional techniques (CV). Twenty disk (3 mm × Ø10 mm) and ten bar specimens (25 × 2 × 2 mm) were fabricated for the CC, 3D, and CV groups, to be used for surface roughness, micro-hardness, and flexural strength testing using standardized protocol. Three indentations for Vickers micro-hardness (VHN) were performed on each disk and an average was identified for each specimen. Surface micro-roughness (Ra) was calculated in micrometers (μm) using a 3D optical non-contact surface microscope. A three-point bending test with a universal testing machine was utilized for assessing flexural strength. The load was applied at a crosshead speed of 3 mm/min over a distance of 25 mm until fracture. Means and standard deviations were compared using ANOVA and post hoc Tukey–Kramer tests, and a *p*-value of ≤0.05 was considered statistically significant. Ra was significantly different among the study groups (*p* < 0.05). Surface roughness among the CC and CV groups was statistically comparable (*p* > 0.05). However, 3D showed significantly higher Ra compared to CC and CV samples (*p* < 0.05). Micro-hardness was significantly higher in 3D samples (*p* < 0.05) compared to CC and CV specimens. In addition, CC and CV showed comparable micro-hardness (*p* > 0.05). A significant difference in flexural strength was observed among the study groups (*p* < 0.05). CC and 3D showed comparable strength outcomes (*p* > 0.05), although CV specimens showed significantly lower (*p* < 0.05) strength compared to CC and 3D samples. The 3D-printed provisional restorative resins showed flexural strength and micro-hardness comparable to CAD-CAM fabricated specimens, and surface micro-roughness for printed specimens was considerably higher compared to CAD-CAM and conventional fabrication techniques.

## 1. Introduction

Interim restorations are critical for the successful management of prosthodontic rehabilitation of natural teeth and dental implants. The term interim means for the time being, pending a definitive arrangement, but this does not imply an expected short-term use. Sometimes, they have to function for extended periods during occlusal equilibration, establishment of occlusal vertical dimension, gingival contour formation around implants, and re-establishment of soft tissues after surgical periodontics. In all these scenarios, the interim restorations can be extremely crucial as they allow the patient to evaluate comfort, function, and appearance prior to the placement of the definitive restorations. Interim restorations must provide pulpal and periodontal protection, esthetic and occlusal stability, marginal integrity, and resistance to functional loads for prosthodontic rehabilitations with long-term clinical success [1,2,3].

Most of the aforementioned requirements regarding interim fixed dental prostheses are influenced by the physical properties of the materials in use, including durability, flexural strength, chemical stability, micro-hardness, surface roughness, and wear resistance [3,4]. Flexural strength represents the resistance of a material against deformation, especially critical for long-span, full-mouth, implant-supported, interim-fixed dental prostheses [5,6]. In addition, surface micro-hardness is a fundamental mechanical property, which is the resistance of a material to plastic deformation typically measured under an indentation load [7,8]. An increasingly rough surface can also promote the initiation of cracks, leading to a shorter restoration life and poor optical characteristics [9]. Adequate hardness ensures that restorations are resistant to surface wear, deformation from mastication, and occlusal abrasion. Higher material micro-hardness increases the wear resistance to a material, which clinically translates to a reduced loss in vertical dimension [7,8,9,10,11]. Moreover, an increasingly rough-surfaced interim can promote bacterial colonization and staining, which is responsible for periodontal inflammation and infection affecting the prognosis of the prosthesis [11]. Therefore, mechanical properties including surface roughness, micro-hardness, and flexural strength are essential for interim restorative materials for better clinical prognosis [1,2,5,6,11,12,13].

Conventionally, interim restorations are fabricated with conventional indirect techniques using polymethylmethacrylate (PMMA) or bis-acryl. These have certain disadvantages, however, including high polymerization shrinkage, exothermic reaction during setting, water sorption, residual monomers, porosity and fractures, color instability, and lack of marginal integrity [14,15]. By contrast, CAD/CAM interim restorations demonstrate significantly lower water sorption, increased wear resistance, adequate micro-hardness, and increased fracture resistance, though it is not free from drawbacks. The initial cost of the equipment and software is high. There is wastage regarding milling burs and restorative materials, and producing complex shapes is difficult [14,15,16]. In addition to this subtractive technique, additive-manufacturing techniques (3D printing and rapid prototyping) have been widely introduced, which fabricate restorations with exceptional mechanical properties and esthetics [17,18].

Aldahian et al. found that 3D-prined specimens had higher surface roughness compared to CAD-CAM and conventional ones [19], whereas Simoneti et al. observed a lower value of surface roughness in the 3D printing specimen compared to conventional specimens [12]. However, a better micro-hardness property in restoration fabricated with conventional techniques rather than 3D printing was reported [12]. Digholkar et al. [6] reported that printed restorations had a micro-hardness value superior to conventional ones. These findings indicate that the existing data on the properties of 3D printing are limited and controversial. Therefore, it is hypothesized that 3Dprinted restorations have better or comparable mechanical properties to CAD-CAM interim resins. It is also hypothesized that 3D -printed restorations will have comparable or better mechanical properties than conventional interim resins. The aim of this study is to assess the surface roughness, hardness, and flexure stress of interim indirect resin restorations fabricated with CAD-CAM (CC), 3D printing (3D), and conventional heat-activated resins (CV).

## 2. Materials and Methods

**Figure 1:**
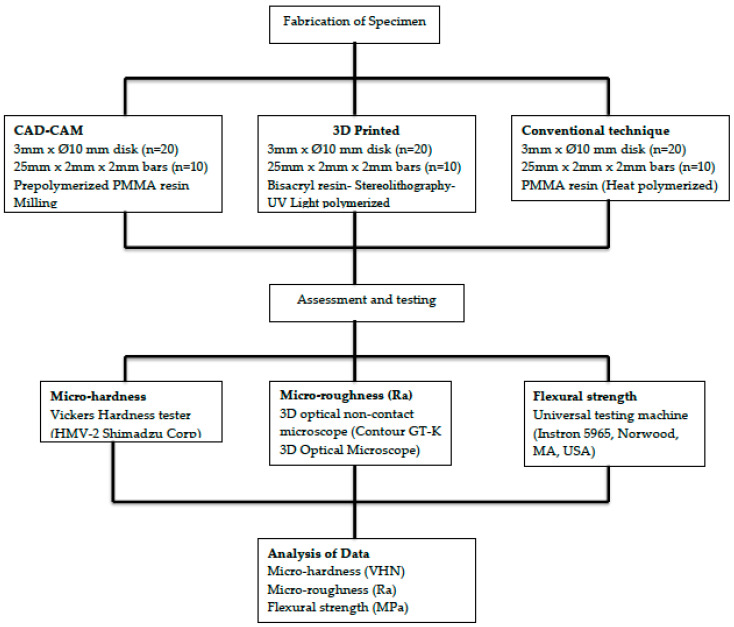
Study methodology.

This study assessed surface roughness, micro-hardness, and flexural strength of interim restoration material samples fabricated with CAD-CAM (CC), 3D printing (3D), and conventional method (CV). Twenty disk specimens with a 3 mm thickness and a 10 mm diameter were fabricated for each group to assess surface roughness and micro-hardness. In addition, ten specimens with dimensions of 25 mm × 2 mm × 2 mm (ISO 4049:2009 7.11.1.1) were fabricated for each group for flexural strength testing. The study outline is presented in Figure 1.

### 2.1. Specimen Fabrication

CAD-CAM specimens were prepared using PMMA resin blanks (Ceramill Temp, shade A1, AmannGirrbach, AG, Koblach, Austria). The specimen design in the stereolithography (STL) file format was transferred to Ceramill Mind (CAD software) and the specimens were milled in a milling machine (Ceramill Motion 2, AmannGirrbach, AG, Koblach, Austria). Standard parameters for milling Ceramill Temp were selected and specimens were fabricated [20]. Specimen finishing was performed according to the milling system recommendations. The 3D-printed specimens were prepared using a stereolithography-based 3D printer (MiiCraft 125; MiiCraft, Jena, Germany) with a light-cure biocompatible resin (Freeprint Temp; DETAX GmbH & Co. KG, Ettlingen, Germany). Using standard printer settings, the STL file specimens were fabricated using CAD-CAM software [21]. After printing, specimens were soaked in 99% isopropyl alcohol (60 s) followed by drying with compressed air. In post-fabrication, the specimens were polymerized with UV light (5 min) in a chamber (220 V; Paul H. Gesswein & Co., Inc., Bridgeport, CT, USA).

For the conventional technique group (CV), specimens were prepared with polymethylmethacrylate (PMMA) material (Jet Tooth Shade™ Self-Curing Acrylic Resin, 6/1 Kit-Lang Dental Manufacturing Co., Inc. Illinois, IL, USA). Using pattern resin (GC Corporation, Tokyo, Japan), specimens of the required dimensions were prepared. The intermediate specimens were inserted in flasks with dental stone to produce molds. The PMMA resin was mixed and prepared according to the manufacturer’s instructions to produce thirty specimens for testing. The PMMA was allowed to polymerize for 10 min and placed in a warm water bath at 55 °C for 10 min.

The standard mechanical properties for the CAD-CAM (Ceramill Temp), printed (Freeprint Temp), and conventionally fabricated Jet Tooth Shade materials are accessible in their respective catalogs [20,21,22]. 

### 2.2. Specimen Testing

For micro-hardness testing, Vickers micro-hardness indentation (VHN) was performed on disk specimens among the three groups (CC, 3D, and CV). Three indentations were randomly made on the surface of each specimen using a Vickers hardness tester (HMV-2 Shimadzu Corp, Tokyo, Japan). Each indentation was separated by 0.5 mm, and 100 g of load was applied for 15 s (Standard-ASTM C1327–03). The three values were averaged to give a single Vickers hardness (VH) for each specimen.

Surface micro-roughness (Ra) was calculated perpendicular to the lay direction in micrometers (μm) using a 3D optical non-contact surface microscope (Contour GT-K 3D Optical Microscope, Bruker^®^, Tucson, Arizona, USA). The device used a laser beam of 2 μm (diameter) and a vertical resolution of 10 nm. The reflected laser light from the specimens’ surface created an image revealing the surface alteration. The collected images were compared and analyzed using software to display surface roughness.

For flexural strength testing, a universal testing machine (Instron 5965, Norwood, MA, USA) was utilized to perform a three-point bending test. A customized jig was prepared to support the bar specimens (25 × 2 × 2 mm) for fracture testing [23]. The load was applied at a crosshead speed of 3 mm/min, until fracture. The fracture load was converted to flexural strength (σ) using the following formula:

σ = 3PL/2wb^2^ (1)

where σ is flexural strength, P is fracture load (N), L is the distance between the two supports (20 mm), w is the specimen height (mm), and b is the specimen width (mm). The flexural strength values were obtained in Mega Pascals (MPa).

### 2.3. Statistical Analysis

The normality of the data was assessed using the Kolmogorov–Smirnov test. Means and standard deviations of micro-hardness, roughness, and flexural strength were tabulated. Comparison of variables was performed using ANOVA and post hoc Tukey–Kramer multiple comparison tests.

## 3. Results

**Figure 2:**
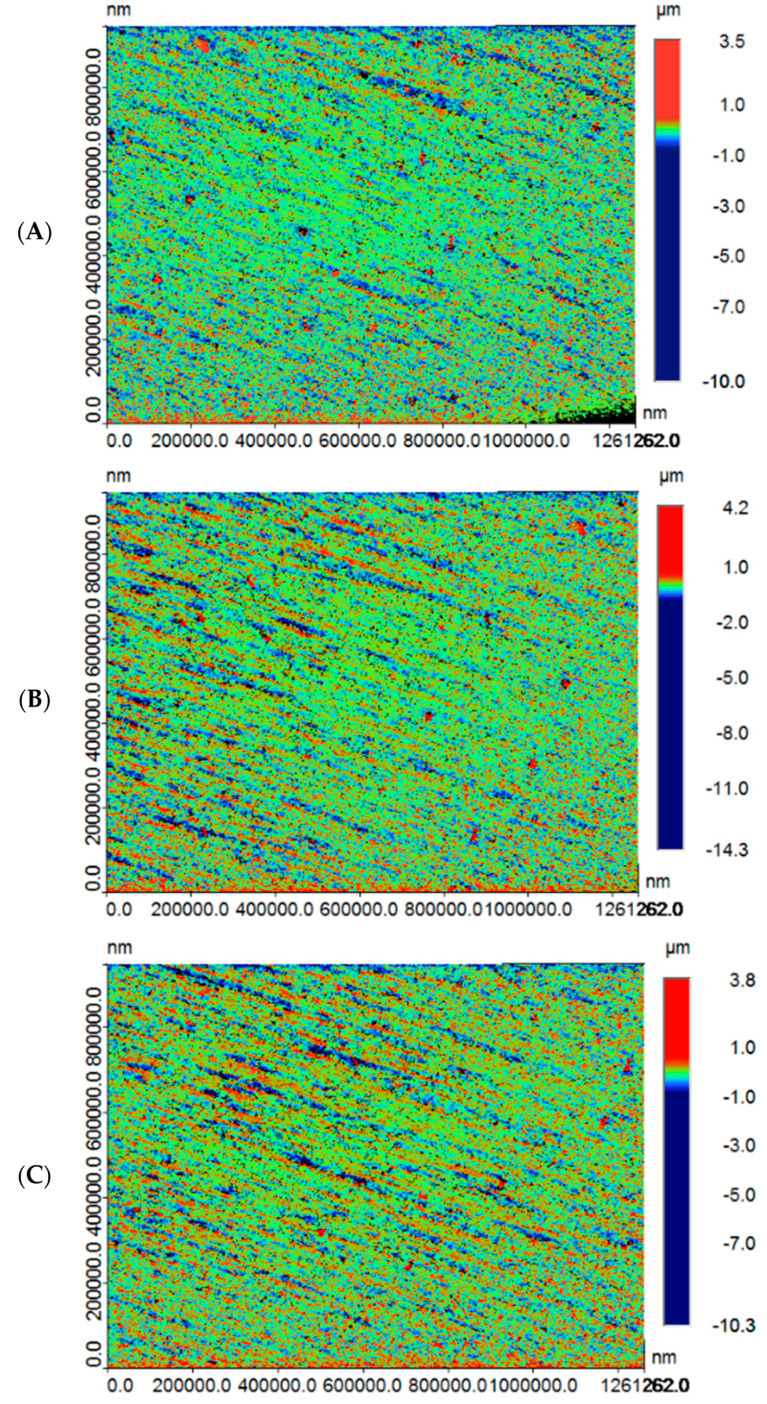
Roughness (Ra) micrographs for study samples in (**A**) CAD-CAM specimen (CC), (**B**) 3D-printed specimen (3D), and (**C**) conventional (CV) specimen groups.

The highest and lowest Ra was observed in 3D (5.77 ± 0.60 μm) and CC (3.68 ± 0.42 μm) specimens, respectively. The mean Ra among the CV specimens was 4.11 ± 1.45 μm (Table 1). The minimum and maximum Ra in CV samples was 2.66 μm and 5.56 μm, respectively. In addition, the highest and lowest Ra for a CC specimen was 3.26 and 4.1 μm, respectively. Among the 3D specimens, the highest and lowest Ra was 6.37 μm and 5.17 μm, respectively. Ra was significantly different among the study groups (*p* < 0.05). Surface roughness among the CC and CV groups was statistically comparable (*p* > 0.05). However, 3D showed significantly higher Ra compared to CC and CV samples (*p* < 0.05). Figure 2 presents surface roughness micrographs for different samples in CC (Figure 2A), 3D (Figure 2B), and CV (Figure 2C) samples.

**Figure 3:**
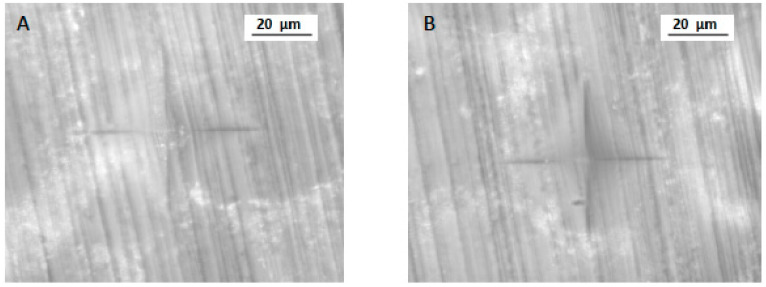
VHN indentation for CAD-CAM samples (group CC). **A** and **B** are two different samples from the same group.

**Figure 4:**
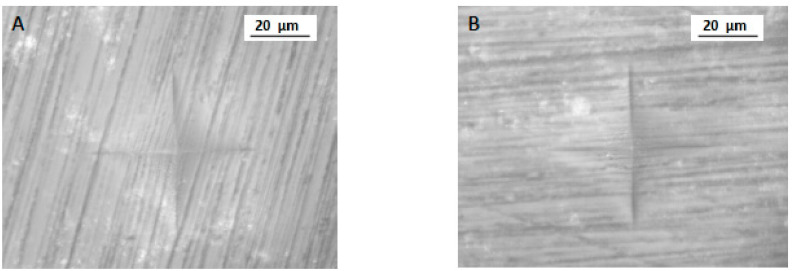
VHN indentation (**A**,**B**) for 3D-printed samples (Group 3D). **A** and **B** are two different samples from the same group.

**Figure 5:**
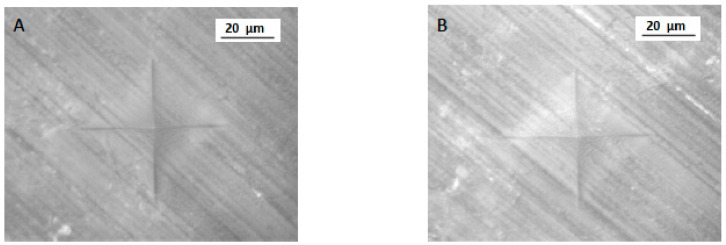
VHN indentation (**A**,**B**) for conventionally fabricated samples (Group CV). **A** and **B** are two different samples from the same group.

For micro-hardness (VHN), the highest and lowest mean was observed in 3D (25.16 ± 4.12 VHN) and CV specimens (21.68 ± 5.53 VHN), respectively. CC specimens showed a mean of 22.07 ± 4.01 VHN (Table 1 and Figure 3). The minimum and maximum VHN among 3D, CC, and CV samples was 21.04 and 29.28; 18.06 and 26.08; and 16.15 and 27.21, respectively. A significant difference was observed in micro-hardness among the study groups (*p* < 0.05). Micro-hardness was significantly higher in 3D samples (*p* < 0.05) (Figure 4) compared to CC and CV specimens (Figure 5). In addition, CC and CV showed comparable micro-hardness (*p* > 0.05).

For flexural strength assessment, CC and CV showed the highest (116.09 ± 13.29 MPa) and lowest means (93.68 ± 17.66 MPa), respectively (Table 2). However, the flexural strength among 3D specimens was 113.16 ± 15.70 MPa. The minimum and maximum strengths among 3D, CC, and CV samples were 97.46 MPa and 128.86 MPa; 102.8 MPa and 129.38 MPa; and 76.02 MPa and 111.34 MPa, respectively. A significant difference in flexural strength was observed among the study groups (*p* < 0.05). CC and 3D showed comparable strength outcomes (*p* > 0.05), although CV specimens showed significantly lower (*p* < 0.05) strength compared to CC and 3D samples.

## 4. Discussion

The present study was based on the hypotheses that 3D -printed restorations have mechanical properties better or comparable to CAD-CAM interim resins; and, secondly, that 3D printed restorations have comparable or better mechanical properties compared to conventional interim resins. In the presented study, printed specimens showed higher micro-roughness, higher micro-hardness, and flexural strength similar to CAD-CAM specimens. Printed specimens also showed higher micro-roughness, micro-hardness, and flexural strength compared to conventional specimens. Therefore, both hypotheses were confirmed. The outcomes observed in the presented study are attributed to differences in material composition, including the type and amount of filler particles, type of curing light for polymerization, processing temperature, 3D-printing parameters, and post-polymerization procedures.

Three-dimensional-printing technology is an emerging tool that uses additive manufacturing to fabricate objects in multiple layers with minimal material waste. Its role in diagnostics and treatment planning procedures in oral care is well-established, and with the development of evolving technology and novel biomaterials, its application in the fabrication of dental restorations has improved [18,19,20]. Surface roughness, micro-hardness, and flexural strength are critical properties for the successful application of interim dental restorations, warranting an investigation of novel 3D-printed interim specimens for clinical use [19]. Vickers micro-hardness indentation (VHN) for micro-hardness testing, Ra assessment for surface micro-roughness (Ra), and a three-point bending test for flexural strength investigation are all standardized and reliable methods for material assessment, allowing comparison of outcomes with previous studies [6,24].

One of the important requirements for an interim restoration is good surface quality. A lower surface roughness value is directly related to biofilm formation, which plays a major role in restoration esthetics and periodontal health [19,25]. In the present study, the highest mean value for surface roughness (Ra) was found in 3D-printed specimens (5.77 ± 0.60 μm), whereas the lowest mean value was observed in CAD-CAM specimens (3.68 ± 0.42 μm). These observations are similar to a recent study showing higher Ra among printed specimens compared to CAD-CAM [19]. Similar findings were also observed in other studies [26]. By contrast, Simoneti et al. [11] reported low surface roughness of 3D-printed specimens compared to conventionally fabricated restorations. This could be attributed to the composition of the material employed, curing light used for polymerization (UV light vs. LED), and the parameters of roughness measurement. According to the published data, different compositions and polymerization times can alter the properties of the resin interims [27,28]. In addition, manufacturing techniques can influence restorative surface roughness, as reported by Arnold et al. [28]. They concluded that roughness values within a certain range could be achieved only under defined circumstances [28]. Moreover, printer parameters such as system type, layer thickness, orientation with respect to building direction, and slicing, impact the mechanical properties of the product [27,28,29,30,31]. In a study by Cheng et al., it was suggested that a combination of inclination and reduced layer thickness (15° inclination; 25 µm) results in a significantly smoother surface [32]. Furthermore, according to Dikova et al. [29], the procedures in currently available 3D printing systems cannot ensure adequate surface quality, and a significant difference in the average Ra was reported when two different 3D-printing technologies were evaluated. Therefore, to fabricate a restoration with optimum surface topography, studies comparing contemporary 3D-printing systems are recommended.

In the present study, a significant difference between the micro-hardness values was observed for all the tested groups. The 3D-printed specimens showed significantly higher micro-hardness than CC and CV specimens. These findings are in accordance with a study by Digholkar et al. [6], where the micro-hardness of 3D-printed specimens was highest between CAD-CAM and conventional samples. The increased micro-hardness value could be attributed to the fact that bis-acryl composite resins used in 3D printed specimens have cross-linked monomers and inorganic fillers, which increase abrasion resistance and decrease polymerization shrinkage [6,33,34]. In addition to surface roughness and micro-hardness, the flexural strength of the specimen was also assessed in the present study. The assessment revealed higher mean flexural strength for CAD-CAM specimens compared to 3D printed specimens, though there was no statistical difference. In a study by Digholkar et al., the CAD-CAM showed higher flexural strength compared to the 3D group interim specimens [6], though the values of printed specimens were inferior to what we found in the present study (113.16 MPa vs. 79.54 MPa). Conflicting results were also observed by Joshi et al. [35]. In the authors’ opinion, the improved flexural strength in printed samples in the present study could be attributed to the process parameters, including build orientation, layer thickness, post-curing, and material composition (printed urethane methacrylate and printed acrylic ester resin vs. light-cure biocompatible resin) [35].

Within the parameters of the study, it was observed that 3D-printed material had better micro-hardness than and comparable flexural strength to CAD-CAM specimens, though the surface roughness was compromised. The findings should be interpreted taking into account that outcomes of in vitro experiments are limited to the materials tested. In addition, the oral environment is complex, with higher dynamic non-axial loads, frequent temperature changes, plaque accumulation, and acidic exposure. Exposing the specimens in the present study to such factors could have produced different outcomes. Therefore, clinical trials comparing 3D-printed interim crowns and fixed partial dentures are recommended to validate the findings of the present study. Additionally, critical properties for interim restorations such as color stability, modulus of elasticity, and wear resistance were not addressed in the present experiment. As resins with different compositions, polymerization duration, and printing techniques may produce interim restorations with altered properties, future studies comparing printing resin materials and methods are advocated.

## 5. Conclusions

In this study, 3D-printed provisional restorative resins showed flexural strength and micro-hardness comparable to CAD-CAM-fabricated specimens, and surface micro-roughness for printed specimens was considerably higher compared to CAD-CAM and conventional fabrication techniques. We conclude the 3D rapid prototyping technology for the fabrication of provisional resin restorations is potentially applicable for clinical use.

## Figures and Tables

**Table 1:** Comparison of surface roughness (Ra) and micro-hardness (VHN) among study groups (CC, 3D, and CV).

	Roughness (Ra)		Micro-Hardness (VHN)	
Study Group	Mean	SD	Mean	SD
CAD-CAM	3.68 ^a^	0.42	22.07 ^a^	4.01
3D	5.77 ^b^	0.60	25.16 ^b^	4.12
Conventional	4.11 ^a^	1.45	21.68 ^a^	5.53
*p*-value	<0.01		<0.01	

**Ra** was measured in micrometers; **VHN** in millimeters; dissimilar superscript; lowercase letters denote statistical significance (*p* < 0.05).

**Table 2:** Comparison of flexural strength among the study groups (CC, 3D, and CV).

Study Group	Mean (MPa)	SD (MPa)	*p* Value
CAD-CAM	116.09 ^a^	13.29	*p* < 0.01
3D	113.16 ^a^	15.70	
Conventional	93.68 ^b^	17.66	

Dissimilar superscript small alphabets denote statistical significance (*p* < 0.05).

## Data Availability

Data of the study are available on request from the corresponding author.
